# Emerging infectious disease and fast-track publication: when public health gets priority over the formality of scholarly publishing

**DOI:** 10.1590/0074-02760160001

**Published:** 2016-05

**Authors:** Claude Pirmez, Adeilton Alves Brandão, Hooman Momen

It has long been acknowledged within the scientific community that in the mid-term future the Earth will undergo important changes beyond those deduced from the past behaviour of known physical phenomena. For example, climate change due to rising temperatures is expected to influence several systems, like ocean levels, wind current and rain regimes. Global travel, urbanisation, biomedical manipulation and intensive agriculture are additional factors affecting natural systems which together in turn influence the diversity of plant, animals, and microorganisms in general. As a result, zoonoses represent 60,3% of emerging infectious diseases, more than 70% of which are caused by pathogens of wildlife origin (Jones et al. 2008). Whatever the action suffered by these subsystems (increase or reduction in species number), the fact is that currently we are unable to predict the exact outcomes of these changes.

This limitation to forecast the future does not prevent us to sketch scenarios for probable events, such as the emergence of unknown and lethal infectious agents. Advances in research of infectious disease and epidemiology have empowered society to respond quickly to this kind of threat, though not so effective as to the point of controlling or completely eliminating a disease. The Ebola epidemics is an example of such an event and its response.

Now we have another threat: the Zika virus (ZIKV) and its vector, the mosquito *Aedes aegypti*. The Director-General of the World Health Organization, on 1 February 2016, declared this to be a Public Health Emergency of International Concern (PHEIC). We can only speculate that the spread of both this virus and vector might be seen as signs of the changes anticipated by climatologists and ecologists. However, one thing is beyond any question or doubt: we have to start new ways to accelerate scientific collaboration, including access to data and exchange of research findings.

Recently, the Forum of Scientific Editors of FIOCRUZ decided collectively to give priority to the publication of papers regarding the Zika epidemic in their journals. In the context of this decision and the declaration of the Director-General of the World Health Organization, we, as the editors of the Memórias do Instituto Oswaldo Cruz (MIOC), have decided to create a section on the MIOC website called “Zika Fast Track”, where research manuscripts relevant to the Zika epidemic will be posted. We have also decided to follow the example of the Bulletin of WHO (Dye et al. 2016) and make the data in these papers freely available for unrestricted use, distribution and reproduction provided that the original work is properly cited as indicated by the Creative Commons Attribution license (CC BY).

All research manuscripts concerning the Zika epidemic, after submission to the Memórias do Instituto Oswaldo Cruz, will be evaluated by an Editor and those considered relevant will be assigned a digital object identifier and posted online in the “Zika Fast Track” section of the Web-site within 24 hours. At the same time, manuscripts will be evaluated by peer review. Data in these manuscripts will thus be attributed to the authors while being freely available for reader scrutiny and unrestricted use, distribution and reproduction provided that the original work is properly cited as indicated by the Creative Commons Attribution license (CC BY 3.0). In papers accepted by the MIOC following peer review, this period of open data within the Zika Fast Track will be mentioned in the final publication. In case of rejection after peer review, authors are free to seek publication elsewhere.

Manuscripts can be submitted via the MIOC website memorias.ioc.fiocruz.br.

*Claude Pirmez* Editor in chief *Adeilton Alves Brandão* *Hooman Momen* Editors
